# Parental experiences of live video streaming technology in neonatal care in England: a qualitative study

**DOI:** 10.1186/s12887-023-03907-4

**Published:** 2023-03-04

**Authors:** Katie Gallagher, Ruby Hayns-Worthington, Neil Marlow, Judith Meek, Kathy Chant

**Affiliations:** 1 Elizabeth Garrett Anderson Institute for Women’s Health, University College London, London, UK; 2 University College London Hospital NHS Foundation Trust, London, UK

**Keywords:** Neonatal care, Technology, Parent, Qualitative

## Abstract

**Background:**

The use of bedside cameras in neonatal units facilitates livestreaming of infants to support parental and family bonding when they are unable to be physically present with their baby. This study aimed to explore the experiences of parents of infants previously admitted for neonatal care and who used live video streaming to view their baby in real-time.

**Methods:**

Qualitative semi-structured interviews were conducted after discharge with parents of infants admitted for neonatal care on a tertiary level neonatal unit in the UK in 2021. Interviews were conducted virtually, transcribed verbatim and uploaded into NVivo V12 to facilitate analysis. Thematic analysis by two independent researchers was undertaken to identify themes representing the data.

**Results:**

Seventeen participants took part in sixteen interviews. Thematic analysis identified 8 basic themes which were grouped into 3 organizational themes: (1) family integration of the baby including parent-infant, sibling-infant, and wider family-infant attachment facilitated through livestreaming, (2) implementation of the livestreaming service including communication, initial set up of the livestreaming service, and areas for improvement, and (3) parental control including emotional, and situational control.

**Conclusions:**

The use of livestreaming technology can provide parents with opportunities to integrate their baby into their wider family and friendship community and gain a sense of control over their baby’s admission for neonatal care. On-going parental education around how to use, and what to expect from, livestreaming technology is required to minimise any potential distress from viewing their baby online.

**Supplementary Information:**

The online version contains supplementary material available at 10.1186/s12887-023-03907-4.

## Background

Admission to a neonatal unit provides particular stress for parents. The normal transition to parenthood is disrupted by concerns over their baby’s health, the adaptation to an unfamiliar environment, and the need for complex conversations with healthcare professionals [1, 2]. Postnatal Depression, anxiety, and Post Traumatic Stress (PTS) are described among parents exposed to the neonatal environment, which may impact parenting and infant behaviours [3–7]. Such outcomes may be exacerbated by restricted family access policies, proximity of the family home to the hospital, the needs of siblings and wider family members, and transfer to another neonatal unit for specialist care [8]. These were particular issues during the COVID-19 pandemic, when parental access was restricted in many hospitals as part of cross infection concerns [9]. The use of digital technology, such as web-based cameras and live video streaming, became one way in which the effects of physical separation on parental bonding could potentially be mitigated [10, 11].

The use of livestreaming technology in neonatal units allow parents to see their baby in real-time through a website which can then be viewed on a digital device. Cameras are placed at the cotside and provide a one-way live stream; the system does not support communication or sound. Studies exploring the impact of livestreaming technology in neonatal units highlight positive parental benefits resulting from reduced feelings of separation, and improved parent-infant bonding [10–16]. These studies have also reported adverse effects on parental wellbeing, however, from parents seeing their baby in distress on the livestream, seeing clinical procedures performed on their baby, or technological difficulties with the technology [10, 11, 15, 16]. The majority of studies are quantitative in nature with few using qualitative methodology to explore the experiences of parents who have used livestreaming technology in depth. Little is therefore known about parental support needs to effectively integrate livestreaming into neonatal settings, particularly in the UK context. As part of a programme of investigation into the introduction of livestreaming in a tertiary level UK neonatal unit, this study aimed to explore parental experiences of using livestreaming technology, to determine how parental support can be enhanced when using this service.

## Methods

A web-based bedside camera service (AngelEye Health, Nashville TN, USA) was introduced into our tertiary level neonatal unit in London in January 2019, prior to the COVID-19 pandemic. There are 28 cots in our unit, receiving around 800 admissions per year. Staff received ward-based teaching on the implementation of the livestreaming technology by clinical and research staff, with parents receiving written information about the system before signing an agreement form for the use of livestreaming webcams to be positioned on their baby’s cotside. During their evaluation, the livestreaming cameras were activated by nurses working with families for 2 h in the morning and 2 h in the evening, with additional personalised ‘viewing’ if parents were unable to be physically present for any reason. Livestreaming was available through a password protected website, accessible through parents’ smartphone or tablet, and shared with extended family/friends at their discretion. The wider programme of study pragmatically explored the impact of livestreaming technology upon nursing workload, staff perceptions and parental experiences, to better understand how the use of novel technology in neonatal care can be enhanced [17]. Parental participation included a questionnaire exploring their attitudes towards the use of livestreaming, and optional qualitative interview. The parental experiences study was introduced to parents by a member of the research team, who were also clinical research nurses, after a minimum admission of, or using the livestreaming technology for, three days (some families were introduced to, and consented to using, livestreaming whilst admitted to labour ward), to allow parents to familiarise themselves with the technology. At the end of the questionnaire was an option to share their experiences further in a qualitative interview. If participants opted to share their contact details, they were contacted by the research team to discuss participation and schedule the interview. All participants provided digital signed informed consent prior to participating in the interviews.

### Qualitative interviews

The semi-structured interview schedule was developed following a review of the relevant literature, followed by content review by neonatal healthcare professionals (supplementary data 1). Virtual interviews were conducted by a female neonatal nurse with training in interview techniques (RHW). Whilst the study was planned prior to the COVID-19 pandemic it was launched during the pandemic, due to the reorganisation of research priorities at the participating NHS Trust. Interviews were scheduled at a time convenient to participants, and within 12 weeks of the infant’s discharge home. Interviews lasted from 30–60 min and were video recorded using Microsoft Teams. Transcripts were downloaded and checked for accuracy against the recording, before anonymising and uploading to NVivo V12 to facilitate data management and analysis. Interviews took place between May–October 2021.

### Participants

Purposive sampling was used to recruit a cohort of parents from our tertiary level neonatal unit from those who had consented to be contacted. Parents had to have a baby admitted for care in the neonatal unit, be over the age of 18, be able to communicate in English, and be eligible for livestreaming use (documented social concerns restricted the use of livestreaming technology in some circumstances). This included families whose infant was critically unwell as currently there is no known information around how livestreaming technology facilitates palliative and/or end-of-life care; we aimed to explore this area in more detail to inform future practice. Participants were invited to be interviewed alone or with a partner, at their discretion. We aimed to recruit around 15–20 parents, based on our experience of data saturation in previous studies [2]. During the study period 41 parents (mothers or fathers) indicated interest in the interviews on the initial questionnaire, however as the study progressed we were unable to contact or schedule a suitable time to discuss further with 23 parents, with one further family volunteering for an interview however declining the use of the livestreaming technology. We continued data collection until data saturation had been reached, and then stopped recruitment. The data presented explores the experiences of parents with livestreaming technology.

### Data analysis

Data were analysed using inductive thematic analysis, searching across all interviews to find repeated patterns of meaning within the data which could provide a detailed understanding of participants experiences and perspectives [18]. Data collection continued until data saturation had been reached; when on reviewing the data no new information was emerging, and thus no further coding would be attained. To increase the validity of the analysis, two researchers (RHW & KG) independently immersed themselves in the data prior to generating initial codes and sub themes, which succinctly represented one concept but were broad enough to find variations within the data [19]. Subsequent broader themes were then generated, prior to researchers jointly reviewing their findings to finalise all themes and their meanings [18, 19].

### Ethical approval

The study was approved by the Health Research Authority and Research Ethics Committee (ID: 20/WS/0155) and the Research and Development Department of UCLH NHS Trust (ref: 134,712). The study is reported following the COREQ reporting guidelines for qualitative research.

## Results

Seventeen participants took part in 16 interviews. One interview had both parents present and one interview represented the mother-father dyad of one infant. (Table 1) None of the parents who volunteered to participate in the interviews were receiving end-of-life or palliative care. Thematic analysis of the transcripts identified 8 sub-themes which were arranged into 3 main themes: (1) family integration (2) livestreaming implementation and (3) parental control. Each theme and the corresponding representative verbatim quotes can be found in Tables 2, 3 and 4.

**Table 1:** Information of the families who participated in the qualitative interviews

Family	Present in interview	Infant Sex	Infant’s gestational age at birth (weeks)
1	Mum	M	38
2	Mum	F	34
3	Mum & Dad	F	30
4	Mum	M	32
5	Dad	M	40
	Mum	M	40
6	Mum	M	30
7	Mum	F	27
8	Mum	M	38
9	Mum	M	37
10	Mum	M	31
11	Dad	F	31
12	Mum	M	33
13	Mum	F	25
14	Mum	M	36
15	Mum	M	31

**Table 2:** basic themes and their respective quotes within the wider organisational theme of family integration (F = family M = mum D = dad)

Organisational Theme: *Family Integration*	Quotes
Basic themes: *Parent-baby bonding*	F8(M):And it's like, no, I have had a baby and it's there. That's my baby. So I just think mentally, psychologically it has loads of benefits. F10(M)**:** Our baby was in a hospital which was about an hour from our house and because we also have a two-year-old toddler it meant we couldn’t be there all the time, or as early as we wanted in the evenings…so when it was mentioned that we could use it (livestreaming), we thought great! Cause then we can see him, then in the evening like say goodnight to him and that would be really sweet. I just thought that’s so great. F1(M): we’d all sit and watch him on a big screen eating breakfast, it was really…social F13(M): I could be pumping and you know, and doing my early morning expressing whilst watching the babies on my phone and I could feel kind of connected to them…that was really really beneficial
*Grandparents / wider family*	F13(M): it was really super positive for them (grandparents) and, lovely for us as well, because you know, even though we could send still pictures, it's not the same as actually kind of getting to know the personalities of the babies and so even though there wasn't the sound, it was lovely for them to be able to interact and see, you know, start to learn the kind of babies personalities a little bit…we had them virtually babysitting, so we'd say, you know, we're having a shower at this point, or you know having some dinner, or, you know, getting to and from the hospital, so could you watch? Have a little look and see how they're doing, so that was really lovely, it was almost like a virtual babysitting service where they could just keep an eye on, in that way F4(M): it was both sets of grandparents and aunties…then there was a very close group of friends that we have…and we’re all really touched that we had offered it to them, and they got to feel connected with baby as well F6(M): they (wider family) used to watch him, even if I’m like, for example, I’m busy with my son…or I shower or something, I’d send the link to my sisters and I’d say, watch they baby until I finish showering, or cooking, or something like that
*Siblings*	F10(M): we got a teddy from home and she (sibling) washed it for him and we took it in and we gave, we put it in his incubator, so that she could see that he had the teddy which was really helpful for her to see, like something she had that he had and kind of helps them, helped her like associate that teddy with the baby and what was going on 'cause it's hard for two year olds to get her head around F11(D): it as really good to have the webcam because, so each night before she (sibling) went to bed, cause the times worked out perfectly for this, she’d get to see baby and she’d give the phone a kiss and a cuddle before going to bed. So it was good for her to kind of get used to the idea of having a brother as well F6(M): when he (sibling) saw him on webcam, he would say oh my god, this is my baby, baby came out of your belly! So happy looking at him and moving his finger and moving his head and stuff like that. Mummy, come, come! He’s moving his finger!

**Table 3:** basic themes and their respective quotes within the wider organisational theme of implementation (F = family M = mum D = dad)

Organisational Theme: *Livestreaming implementation*	Quotes
Basic themes: *Communication*	F1(M): I was made aware that you know, if there's a doctor that needs to do something, the camera will be switched off or it will be turned away. So I did more often than not, see peoples feet and things, but I think I…it was all explained to me. I felt as though I was given, like, as soon as we signed up for the camera, I felt as though…the information sheet that you gave and, and also the nurse explained everything to me. So nothing came as a shock F12(M): (there is) a difference between the stress in person and the stress on the camera…because we couldn’t hear her, we didn’t know just how much, you know, distress she was in…in real life she might have just been going ahh and stretching but then on the camera, it looked like she was squealing in anguish, so we didn’t know what was going on F6(M): it’s always if his hand is in his face and the wires around his neck, I would be worried…and that is just worrying, until I came down after the 10 days (COVID isolation) and I saw the process and the nurses explained to be it’s just a wire around his chest that’s on his neck, and we’re seeing him and there is like the machines, I didn’t know all about that
*Initial set up*	F3(M): We were told about the camera quite late like the third week…so she was in intensive care for two weeks and we never were suggested about that. And a Doctor at the third week in special care was making rounds and he told me…because he was the neonatal Doctor that got baby like when she was delivered. He took pictures of baby and sent them to print with someone and sent it to me, to have it with me when I went to postnatal ward and that was very helpful. I was grateful for that and I told him and he told me like yes, and that's why we have the cameras for watching the babies and I was like what cameras…I don't know about the cameras, and then we started F2(M): There was a technical difficulty at the very beginning and so I think I signed a form whilst I was still on labour ward and I was in a bit of, like a haze, so I don’t know if it was my fault or but then my email, either I’d not written it down right or it hasn’t been inputted right so we didn’t actually get it set up for a week
*Areas for improvement*	F2(M): Somehow, if you made that sort of electronic or something and you could check, you know… Make sure it’s written correctly or something (email address). I didn't quite understand how it worked, 'cause I did follow up when, when I was on ITU, I did follow up and say, you know is the angel eye working and they just say oh you should get an email, should get an email, but I obviously didn't because it was typed wrong, so, um it just took a few days to kind of, you know log in and for it to work F13(M): the nurses were always really lovely and tried sort it out (livestreaming technical problems), but I think the problem is that they didn't always know. Not all the nurses had the expertise around it. I think different nurses have different levels of knowledge of how to, you know, problem solve, if there was an issue

**Table 4:** basic themes and their respective quotes within the wider organisational theme of parental control (F = family M = mum D = dad)

Organisational Theme: *Parental control*	Quotes
Basic themes:*Emotional (themselves & others)*	F8(M): Who do you give it to (login details for the live feed)? Do you give it to my mum and dad, do you give it to his mum and dad, my siblings. I just, you know, in, in that kind of time it's so stressful, and I didn't really want 1000 questions. What's that on her? And what's that wire about? Why those stickers on a chest? You know those kind of things are simple questions, but when you’re going through something so stressful, you just don't want hear it F1(M): I had a (family member) that was pregnant and I thought it would be wrong of me, you have to be a bit sensitive I guess, (baby) was all wired up and it might not have been pleasant to see…he was my baby and regardless of what he looked like, I wanted to see him, it might not have been pleasant for other people F3(D): Some days I would just be working and log in for a few minutes to see her specially before mum arrives, so she would leave our place and there will be like, I don’t know, an hour or half an hour window where until there she wouldn’t be there, but I would. Mum would not be in the hospital yet and then sometimes I would log in and check her. And yeah, it was reassuring in a way
*Situational*	F2(M): I had a little bit of a long commute…to the hospital…I sort of want to get there really quickly to make sure she was ok, but actually I could log in on the train…I could just see, oh no, it’s fine, she’s alright F5(M): Then you’ve come in and you’ve got one member of staff, they’re doing something important and you think, ah I wish I could switch that camera on, I wish there was a button because I could turn it on and turn it off as I see fit! It’s my baby, what do you want people to see? I don’t need you to do it, I can flick it on, flick it off….the nurse that running that unit…she gotta get that baby fed, that one fed, boom boom boom, temperature checks…so you’re not gonna ask, you’re not gonna ask for the camera, I’m not anyway. We’re not going to ask F5(D): the only issue would be whether they were going to put the camera on so you know, my wife would phone up sometime the nurses and ask them to put it on and to a certain extent you know there may be an awkwardness about asking them to do it, because you can see they’re very busy

### Family Integration

The first theme ‘family integration’ represented 3 sub-themes: (1) parent-infant attachment, (2) sibling-infant relationship, and (3) wider family-infant relationship. Parents discussed how livestreaming broke down barriers within their family, allowing shared conversations about their baby and their progress, and facilitating cohesion of the family unit. Parents reported greater opportunity to feel more involved in their baby’s care, particularly during absence for work or childcare commitments. Parents described the importance of increased sibling and wider family involvement through livestreaming, allowing for relationships to develop and providing familiarity of the baby within the family.

### Livestreaming Implementation

The second theme ‘livestreaming implementation’ represented 3 sub-themes: (1) communication, (2) initial set up, and (3) areas for improvement. Parents highlighted a need for increased communication to manage expectations, prepare them for potential adverse events and how livestreaming was integrated into ward routine. Parents discussed issues around livestreaming set-up and on-going technical challenges, and their perceived difficulties in addressing these on the unit. Parents discussed ways in which livestreaming could be improved, including a more parent-led approach, and staff training to assist with technical issues and trouble shooting.

### Parental Control

The final theme ‘parental control’ represented 2 sub-themes: (1) emotional control, and (2) situational control. Parents reported using livestreaming as a way of controlling their anxiety when they could not be physically present with their baby, for example before bed and/or first thing in the morning, to reassure themselves their baby was ok. This need to see their baby, however, was fraught with worry as parents reported concerns they may see something ‘wrong’ with their baby during the livestream. Parents discussed their need to control the emotional impact upon family and friends, through restricting others viewing of their baby. This simultaneously provided parental self-protection from the reactions of others, in times when parents were already highly stressed. Parent expressed mixed emotions around the situational control livestreaming provided, from allowing them to plan daily activities with less pressure, knowing they can still see their baby, to frustrations with technical issues, and lack of autonomy over livestreaming control.

## Discussion

Through qualitative interviews this study was able to explore parental experiences of using livestreaming technology on a tertiary level neonatal unit. Thematic analysis of seventeen semi-structured interviews identified 3 organisational themes highlighting the importance of family integration, livestreaming implementation, and parental control. Our findings reinforce previous research suggesting livestreaming technology can promote emotional attachment in families [14, 15], with parents in our study reporting a sense of connection with their baby when they cannot be physically present. This connection appears to be enhanced by the ability to integrate their baby into their wider family and friendship community, creating a shared connection through loved ones getting to know their baby [10, 20]. In our study, as with others, the ability of livestreaming to facilitate integration of siblings was reported to be helpful, particularly for those with younger children, who may be confused about where the new baby is after their mother returned from hospital after giving birth [20]. Parents joy at seeing their baby through livestreaming, however, was often tempered with anxiety as to what they may see (e.g. their baby crying, an adverse event) and/or technical difficulties initiating or accessing the livestream.

These concerns have also been identified among healthcare professionals using livestreaming technology. Kubicka et al. found that 70% of neonatal staff surveyed reported they were concerned parents may witness events on the livestream which may be distressing [21]. These findings indicate the importance of healthcare professionals introducing livestreaming thoroughly to parents prior to their use, describing daily neonatal unit activities and what they may see on the camera in detail. This could include the equipment surrounding their baby, cares / examinations they may observe, and what would happen in the event of an ‘emergency’, both with the treatment of their baby and the livestream. Ensuring parents are properly informed could allow them to manage their own (and others) expectations of livestreaming and facilitate conversations around what parents would like to see on the livestream. Studies have identified that while cameras may be switched off during routine cares or procedures, in our study, parents reported they enjoyed seeing these activities, and enhanced their feeling of involvement in their baby’s care [11, 13]. Clearer communication between parents and professionals is therefore required to determine when the web cameras will be livestreaming (if not 24 h a day), and what activities will be livestreamed, to minimise parental distress and facilitate engagement with their baby.

As with other studies, parents reported livestreaming facilitated a sense of control, providing reassurance when needed that their baby was well, and facilitating necessary activities outside of the neonatal unit [10, 11, 14, 22]. Some parents reported frustration from loss of this control through technological difficulties when using livestreaming, or through not being able to control the camera timings (camera timings were limited to two hours per morning and evening, respectively). The inability to personally manage the livestreaming service may compound a wider sense of parental loss of control often reported by parents whose infant is admitted for neonatal care [23]. There is a current drive in our own unit, as with many others, to involve parents to a greater extent than previously in their baby’s care, for example through Family Integrated Care (FIC), which incorporates families as members of the care team as a way of supporting them to become actively engaged in their baby’s care [24, 25]. As an approach, FIC has been reported to have positive impact by reducing parental anxiety at discharge, and the baby’s weight gain through the admission. In turn this is anticipated to have effects on long term infant development through improved parental bonding and mental health [25]. As the use of livestreaming has the potential to enhance a parents sense of connection with their baby, simultaneously parental control over the livestreaming itself may facilitate further integration into the neonatal care team and FIC, allowing parents to create a livestreaming schedule tailored to their individual family needs.

Whilst the integration of livestreaming technology could potentially enhance parental engagement with their baby, it creates additional training needs for healthcare professionals. Studies exploring staff perceptions of livestreaming identified a common distrust of the technology, concerns about impact on workload, and wariness of being ‘watched’ on camera [11, 20, 26]. Whilst there is little research exploring the perceptions of neonatal professionals towards the proliferation of technology in neonatal settings, research conducted with adult intensive care healthcare professionals identified various barriers to implementing new technology including the demand for more time, negative impact upon workload, lack of education and complexity of the technology itself [27]. The integration of digital technology into patient care must therefore be supported by continued investment in professional development opportunities, for staff to improve their confidence and competence in their digital skills [28, 29]. The potential for future specialist digital roles designed specifically to support the use of enhanced technology in neonatal settings is something that could be considered in future, to support both healthcare professionals and parents to optimise their care of the baby.

### Limitations

Our study provides the perspective of only 17 participants from one neonatal unit, which may not be reflective of parents in neonatal units in general. The study was also planned prior to the COVID-19 pandemic but undertaken during periods of lockdown, which may have influenced parents’ decision to consider livestreaming use. Interviews were undertaken by a neonatal nurse researcher who had provided clinical care for some of the participating parents, who may therefore have been more reserved in their responses. Our study, however, is one of only a few which has explored parents’ experiences of livestreaming technology in the UK. Our findings provide insight which can help to improve parental support during their baby’s stay on the neonatal unit.

## Conclusion

Our findings highlight that parents report opportunities provided by livestreaming technology to integrate their baby into their wider community, and to gain an improved sense of control and reassurance over their situation. Parents also highlight important issues around communication of how to use, and what to expect from, livestreaming, to minimise any adverse parental distress. These findings have implications for healthcare professionals in relation to their initial and on-going education of parents in their baby’s care, and their professional digital skills. Addressing these areas could improve the future use of livestreaming technology in neonatal units for families and professionals.

## Supplementary Information

**Additional file 1: Supplementary information 1. **Livestreaming technology parent interview topic guide

## Data Availability

The datasets generated during and/or analysed during the current study are available from the corresponding author on reasonable request. Ethics approval and consent to participate. The study was approved by the Health Research Authority and Research Ethics Committee (ID: 20/WS/0155) and the Research and Development Department of UCLH NHS Trust (ref: 134712). All participants provided digital signed informed consent prior to participating in the interviews. All methods were carried out in accordance with relevant guidelines and regulations.
