# Supplementary material for: Parental experiences of live video streaming technology in neonatal care in England: a qualitative study
Source: BMC Pediatr. 2023 Mar 4;23:107. doi: 10.1186/s12887-023-03907-4 (PMC9984744; doi:10.1186/s12887-023-03907-4)
Supplement: Supplementary file 1 — Additional file 1: Supplementary information 1. Livestreaming technology parent interview topic guide [file 12887_2023_3907_MOESM1_ESM.docx]

**Supplementary information 1: Livestreaming technology parent interview topic guide**

*Prior to signing up*

What was it about the webcam made you decide to use it?

Did you have any concerns about using webcams on your baby?

*Using webcams on the neonatal unit*

Did you experience any times when you, your partner or extended family could not visit your baby when you wanted to, due to COVID-19?

What impact did the webcam have during these situations?

When do you mostly log on to see your baby?

- Is there a particular time of day?
- How often do you use it?

Have you shared access to the webcam with anyone else?

- Who and why did you share access?
- How did you find having others being able to see your baby on screen?
- How did they find being able to see your baby on screen?

What have you found useful about using the webcam to see your baby?

How did webcams affect your relationship with the nurses and doctors caring for your baby?

What did you find difficult/challenging about using the webcam?

Is there anything you would have done differently had you known what you know now?

Is there anything that you would tell the nurses and doctors around how they can help families using webcams with their baby?

*Future use of webcams*

If you were to give advice to a new parent on the unit regarding Angel Eye – what would you advise?
